# The Use of a Smartphone App and an Activity Tracker to Promote Physical Activity in the Management of Chronic Obstructive Pulmonary Disease: Randomized Controlled Feasibility Study

**DOI:** 10.2196/16203

**Published:** 2020-06-03

**Authors:** Claire L Bentley, Lauren Powell, Stephen Potter, Jack Parker, Gail A Mountain, Yvonne Kiera Bartlett, Jochen Farwer, Cath O'Connor, Jennifer Burns, Rachel L Cresswell, Heather D Dunn, Mark S Hawley

**Affiliations:** 1 School of Health and Related Research The University of Sheffield Sheffield United Kingdom; 2 School of Education The University of Sheffield Sheffield United Kingdom; 3 School of Human Sciences The University of Derby Derby United Kingdom; 4 Manchester Centre for Health Psychology The University of Manchester Manchester United Kingdom; 5 Library The University of Manchester Manchester United Kingdom; 6 Sheffield Teaching Hospitals NHS Foundation Trust Integrated Community Care Team Sheffield United Kingdom

**Keywords:** mobile health, mHealth, chronic obstructive pulmonary disease, feasibility, physical activity, activity tracker, Fitbit, self-management, health behavior change, pulmonary rehabilitation

## Abstract

**Background:**

Chronic obstructive pulmonary disease (COPD) is highly prevalent and significantly affects the daily functioning of patients. Self-management strategies, including increasing physical activity, can help people with COPD have better health and a better quality of life. Digital mobile health (mHealth) techniques have the potential to aid the delivery of self-management interventions for COPD. We developed an mHealth intervention (Self-Management supported by Assistive, Rehabilitative, and Telehealth technologies-COPD [SMART-COPD]), delivered via a smartphone app and an activity tracker, to help people with COPD maintain (or increase) physical activity after undertaking pulmonary rehabilitation (PR).

**Objective:**

This study aimed to determine the feasibility and acceptability of using the SMART-COPD intervention for the self-management of physical activity and to explore the feasibility of conducting a future randomized controlled trial (RCT) to investigate its effectiveness.

**Methods:**

We conducted a randomized feasibility study. A total of 30 participants with COPD were randomly allocated to receive the SMART-COPD intervention (n=19) or control (n=11). Participants used SMART-COPD throughout PR and for 8 weeks afterward (ie, *maintenance*) to set physical activity goals and monitor their progress. Questionnaire-based and physical activity–based outcome measures were taken at baseline, the end of PR, and the end of maintenance. Participants, and health care professionals involved in PR delivery, were interviewed about their experiences with the technology.

**Results:**

Overall, 47% (14/30) of participants withdrew from the study. Difficulty in using the technology was a common reason for withdrawal. Participants who completed the study had better baseline health and more prior experience with digital technology, compared with participants who withdrew. Participants who completed the study were generally positive about the technology and found it easy to use. Some participants felt their health had benefitted from using the technology and that it assisted them in achieving physical activity goals. Activity tracking and self-reporting were both found to be problematic as outcome measures of physical activity for this study. There was dissatisfaction among some control group members regarding their allocation.

**Conclusions:**

mHealth shows promise in helping people with COPD self-manage their physical activity levels. mHealth interventions for COPD self-management may be more acceptable to people with prior experience of using digital technology and may be more beneficial if used at an earlier stage of COPD. Simplicity and usability were more important for engagement with the SMART-COPD intervention than personalization; therefore, the intervention should be simplified for future use. Future evaluation will require consideration of individual factors and their effect on mHealth efficacy and use; within-subject comparison of step count values; and an opportunity for control group participants to use the intervention if an RCT were to be carried out. Sample size calculations for a future evaluation would need to consider the high dropout rates.

## Introduction

### Chronic Obstructive Pulmonary Disease

Chronic obstructive pulmonary disease (COPD) is one of the most prevalent chronic conditions (CCs), and one of the leading causes of death and disability, in the United Kingdom [1] and worldwide [2]. COPD is characterized by progressive and nonreversible narrowing or inflammation of the airways or alveoli in the lungs [3,4]. People with COPD experience symptoms such as breathlessness, frequent chest infections, reduced ability to exercise, and impaired day-to-day functioning [5-7]. COPD and its treatment cost the UK’s National Health Service (NHS) approximately 800 million pounds annually [3,5].

### Self-Management

Even with appropriate medical care, people with COPD experience symptoms and functional challenges on a daily basis, and therefore, they must engage in long-term self-management to maintain their physical, social, and psychological health [8,9]. Teaching people with CCs to self-manage their health has become an important strategy for alleviating symptom burden and improving quality of life and is advocated within the NHS as a method of empowering people to take control of their health [10]. Participation in self-management activities requires changes to personal health behaviors by the individual with COPD. For example, COPD is associated with low physical activity levels [11]—higher levels of physical activity in the management of COPD are associated with a lower risk of hospital admission, a lower risk of COPD-related and all-cause mortality, and a higher health-related quality of life compared with those with lower activity levels [12,13].

### Pulmonary Rehabilitation

Pulmonary rehabilitation (PR) is an intervention that aims to foster self-management among people with COPD in the United Kingdom. It is a group-based program that takes place over a minimum of 6 weeks and aims to teach people with COPD (and other lung conditions) to self-manage their condition [5,14]. UK National Institute for Health and Care Excellence 2018 guidelines [14] stipulate that the service should be available nationally for all people with COPD who have recently been hospitalized with the condition or who are functionally restricted by the condition. The program includes physical exercise of the upper and lower extremities, education about different aspects of the condition and how to manage them (eg, breathlessness), and strategies for improving daily functioning [4,5,14]. PR has demonstrated a number of benefits for people with COPD, including improved exercise capacity, alleviation of symptoms, reduced number and severity of exacerbations, reduced depression, and improved quality of life and sense of control [15]. In addition, PR has been demonstrated as cost-effective in the UK context [5]. However, long-term maintenance of increased physical activity and self-management behaviors after PR completion is a significant challenge [16,17].

### Health Behavior Change

It is advantageous to design health behavior change interventions that are underpinned by behavior change theories or models, eg, the Behavior Change Wheel (BCW) [18]. Within the BCW model, intervention functions and policy categories are arranged around a central *hub* that outlines three different sources for health-related behaviors: opportunity, capability, and motivation. By providing education, training, persuasion, and environmental restructuring and enablement, PR can increase the social and physical opportunity for patients with COPD to engage in self-management, increase the physical and psychological capability of patients to engage in self-management, and help patients feel motivated to carry out self-management behaviors.

The potential for digital technology to help people with COPD to self-manage their condition is increasingly being investigated [19,20] and advocated in the NHS [10,21]. Bartlett et al [22] demonstrated that people with COPD find support with a primary task, and dialog support (eg, through feedback), to be persuasive technological strategies to help increase their levels of physical activity. Therefore, a technological intervention for self-management that incorporates these elements could increase an individual’s capability, opportunity, and motivation to carry out that behavior (eg, motivation through encouraging feedback).

### Mobile Health

Although the exact definition is disputed, mobile health (mHealth) broadly refers to medical or health care interventions delivered through mobile technology (eg, smartphones) [23]. Digital technologies such as mHealth offer several advantages over more traditional forms of care, including low up-front cost [24]; familiarity and convenience for patients [21]; better access to information [24]; improved communication between patients and health care professionals (HCPs) [21,24,25]; provision of real-time feedback to patients [24]; and allowing patients to monitor their own data [24,25]—all of which potentially increase health service efficiency and reduce costs [21,25]. Although mHealth tools represent a promising means to encourage greater self-management of COPD, findings from a systematic review in this field were inconclusive owing to a high risk of bias in the included studies, thus indicating the need for more research [19]. Digital health interventions should be evidence based, person based, and robustly evaluated, with considerations given to future implementation of the intervention at an early stage of its development [20].

### Intervention Development

We developed an mHealth-based self-management intervention for COPD. According to the Technology Acceptance Model, the perceived usefulness of a technological intervention directly affects an individual’s intention to use the technology in question [26]. Therefore, we carried out a large amount of exploratory work with the intended users and stakeholders of the technology. Qualitative semistructured interviews were conducted with people with COPD (n=15), their family members (n=5), and HCPs who work in PR services (n=7). During the interviews, we explored participants’ experiences of COPD self-management, their priorities for self-management, and their views on using digital technology to aid self-management. We also showed participants examples of ways in which digital technology could enhance capability, opportunity, and motivation [18] for self-management of COPD, eg, through goal setting and automatic monitoring of goals, demonstrating different types of devices and their functions, etc. The purpose of these interviews was to explore participants’ reactions to the possibility of using digital technology to help with self-management of the condition and to feed into the design of such an intervention.

During these exploratory interviews, both people with COPD and HCPs identified physical activity as being a high priority for COPD self-management. Feedback from the interviews informed the development of a prototype intervention that used an activity tracker and a smartphone app to help people with COPD set physical activity goals and monitor their progress. A total of 5 in-house researchers (unconnected with the project) and 5 participants with COPD from the exploratory interviews were later shown the prototype intervention and were asked to carry out *think-aloud* tasks using the technology, in accordance with a user-centered design [27]. The 5 participants with COPD involved in this stage of usability testing were selected to include a range of ages, gender, and COPD severities. The usability testing helped with assessing the intervention’s relevance and usability, identified problems in its operation, and helped further refine the intervention. After this stage, 2 people with COPD used the intervention over a period of many weeks. They relayed their experiences of using the intervention and offered suggestions for improvement.

The results of these interviews, of usability testing, and of a scoping literature review of existing best practice guidelines informed the development of a Self-Management supported by Assistive, Rehabilitative, and Telehealth technologies-COPD (SMART-COPD) app for COPD self-management and informed strategies for its use. In addition to the emphasis on physical activity, the importance of HCP support in the path to self-management was also emphasized. Therefore, the intervention was incorporated within the PR program, with the aim of encouraging individuals to maintain increased levels of physical activity after completing PR. In accordance with the BCW approach, the app provides motivation to self-manage physical activity through personalized feedback along with PR and provides the capability and opportunity to continue self-managing physical activity after PR. The development of the mHealth intervention is summarized in a short YouTube video [28].

### Feasibility and Acceptability

Feasibility studies are carried out before large-scale studies (such as randomized controlled trials, RCTs) with the aim of establishing whether an intervention can be used and, if so, how it should be used [29]. The feasibility stage includes testing the intervention for its acceptability, estimating likely recruitment rates and retention of participants, and testing out design elements of a larger study [30]. A mixture of quantitative and qualitative methods is likely needed to establish feasibility [30].

An important element of the feasibility of carrying out a larger study is the acceptability of the intervention itself. In this study, we assessed the acceptability of the intervention using a combination of both qualitative (eg, interview data) and quantitative (eg, usage data collected by the intervention) data. Assessment of acceptability included attitude toward the intervention; burden of the technology; perceived effectiveness of the intervention; how well the intervention fits with the participants’ perceived value system; intervention coherence; and self-efficacy in being able to use the intervention [31]. Although objective data, such as dropout rates, provided a quantitative indication for ease of use of the intervention, the research team did not set predetermined thresholds for levels that would deem the intervention *feasible* or *not feasible*. The study evaluated the feasibility of delivering a complex intervention in a complex health care setting; therefore, qualitative interview feedback and reasons for withdrawal were necessary to understand the nuances behind the intervention’s feasibility.

### Research Questions

This paper reports the results of a randomized feasibility study. The research questions (RQs) were as follows:

- RQ1: Is it feasible and acceptable to use the SMART-COPD intervention within PR to encourage people with COPD to maintain (or increase) their physical activity levels after PR?
- RQ2: Is it feasible to conduct a future large-scale RCT to investigate the effectiveness of the intervention?

Specifically, the following questions were addressed:

- How do people with COPD and HCPs react to the technology? What are their views on the technology, and on whether it is feasible to use the technology for physical activity in COPD? (related to RQ1)
- Is the technology acceptable to people with COPD and HCPs? For example, do they use the technology as intended? Do they find the technology easy to use? Which parts of the technology do they use? Are there any problems with the technology? (RQ1)
- What are the recruitment and dropout rates for the study? What do these patterns tell us about the acceptability of the technology and the feasibility of conducting a future RCT? (RQ1 and RQ2)
- Which outcome measures should be used for a larger-scale evaluation of the technology? (RQ2)
- How do people with COPD react to randomized assignment and to being in the *Control* group? (RQ2)
- How should the technology be deployed: both within health care services and within an RCT? Are any changes needed to increase the feasibility of using technology in this way? (RQ1 and RQ2)

The overall aim of the study was to determine the feasibility and acceptability of both the intervention and the possibility of carrying out a future RCT.

## Methods

### The Self-Management Supported by Assistive, Rehabilitative, and Telehealth Technologies-Chronic Obstructive Pulmonary Disease Intervention

The SMART-COPD intervention is an Android smartphone mobile app used in conjunction with a Fitbit wearable activity tracking device. The app encourages physical activity through goal setting, self-monitoring, and feedback, based on behavior change and persuasive technology principles [18,22]. The app includes three elements of physical activity: general cumulative activity over each day (ie, step count); a timed daily walk; and timed daily exercises based on standard PR exercises (eg, leg lifts). The integrated content of the app is summarized in Table 1.

**Table 1:** Summary of physical activity components within the SMART-COPD intervention.

Facet	Step-count (activity tracker and app)^a^	Daily walk (app only)^a^	Exercises (app only)^a^
Goal type	Number of steps (measured by activity tracker)	Length of walk in minutes (measured by phone's accelerometer)	Length of time exercising (measured via manual timer on phone)
Feedback	Daily step-count visible on activity tracker, and feedback graphs on phone	App shows flower gaining petals as they get closer to their goal, and feedback graphs on phone	Videos demonstrating different exercises, timed doing exercises, and feedback graphs on phone Videos developed in-house with PR physiotherapists and featuring a range of COPD^b^ severities

^a^Individualized goals set in partnership with PR staff. Participants self-monitor progress via described outlets.

^b^COPD: chronic obstructive pulmonary disease.

Participants decided on a personal starting goal for each of the three activities based on advice from their PR team and their own preferences. It was important that participants felt *ownership* of their goals and that both the participant and HCP felt they were achievable. The intention was that they should consistently achieve their activity goals on a daily basis and, if possible, gradually increase each physical activity goal over time. The app was used initially in conjunction with the PR program, with continued use once the PR program had finished.

Study participants were provided a Motorola smartphone with the SMART-COPD app installed and a Fitbit activity tracker for the duration of the study. Each smartphone was fitted with a SIM card to enable remote data transfer. The app could be used in conjunction with 1 of 3 Fitbit models: Charge HR (wrist-worn); Charge 2 (wrist-worn); or One (hip-worn). Fitbit activity trackers use low-energy Bluetooth to automatically transmit (or *sync*) data periodically whenever the device is in the proximity of a smartphone with an appropriately configured Fitbit app. These data are then transferred to Fitbit’s internet servers, where they can be accessed in the SMART-COPD app. The devices have a rechargeable battery with an approximately 5-day battery life. Fitbit One had previously demonstrated high accuracy compared with other low-cost activity trackers even for slower walking speeds (a factor highly relevant for COPD) [32]. The decision to use Fitbit as a step count device was further supported by in-house comparisons of step count accuracy of various activity monitoring devices (including Fitbit One and Charge HR), at different walking speeds, carried out by researchers and people with COPD.

### The Setting

In total, PR teams at 3 NHS sites in Northern England, United Kingdom, participated in the feasibility study. Northern England has one of the highest rates of lung disease in the United Kingdom, possibly due to greater socioeconomic deprivation and higher rates of smoking compared with the south of England [3,4,33]. Preliminary work was conducted to map current PR care pathways (eg, workshops with PR staff; observation of PR sessions, numbers, and demographics of referred patients; etc), and we worked together with the PR teams to determine how the intervention might best be used within the PR program. Each PR service was delivered over a 6- to 7-week timeframe, with similar exercises and educational content. Therefore, the same study procedure was used across all sites.

### Study Design

The study was a randomized feasibility study [34] using both quantitative and qualitative methods. The Medical Research Council Framework for the evaluation of complex interventions emphasizes the feasibility stage as a means of testing procedures and estimating parameters for a future large-scale evaluation [30].

### Participants

The aim was to recruit 30 individuals who were formally diagnosed with COPD and who were attending PR in 1 of the 3 study sites. This sample size was chosen based on advice from an in-house statistician and on a study by Julious [35]. Potential participants met the inclusion criteria if they were attending PR. There were no exclusion criteria based on age, comorbidities, or having previously attended PR for managing COPD. Participants did not need any previous experience of using digital technology.

### Procedure

**Figure 1:**
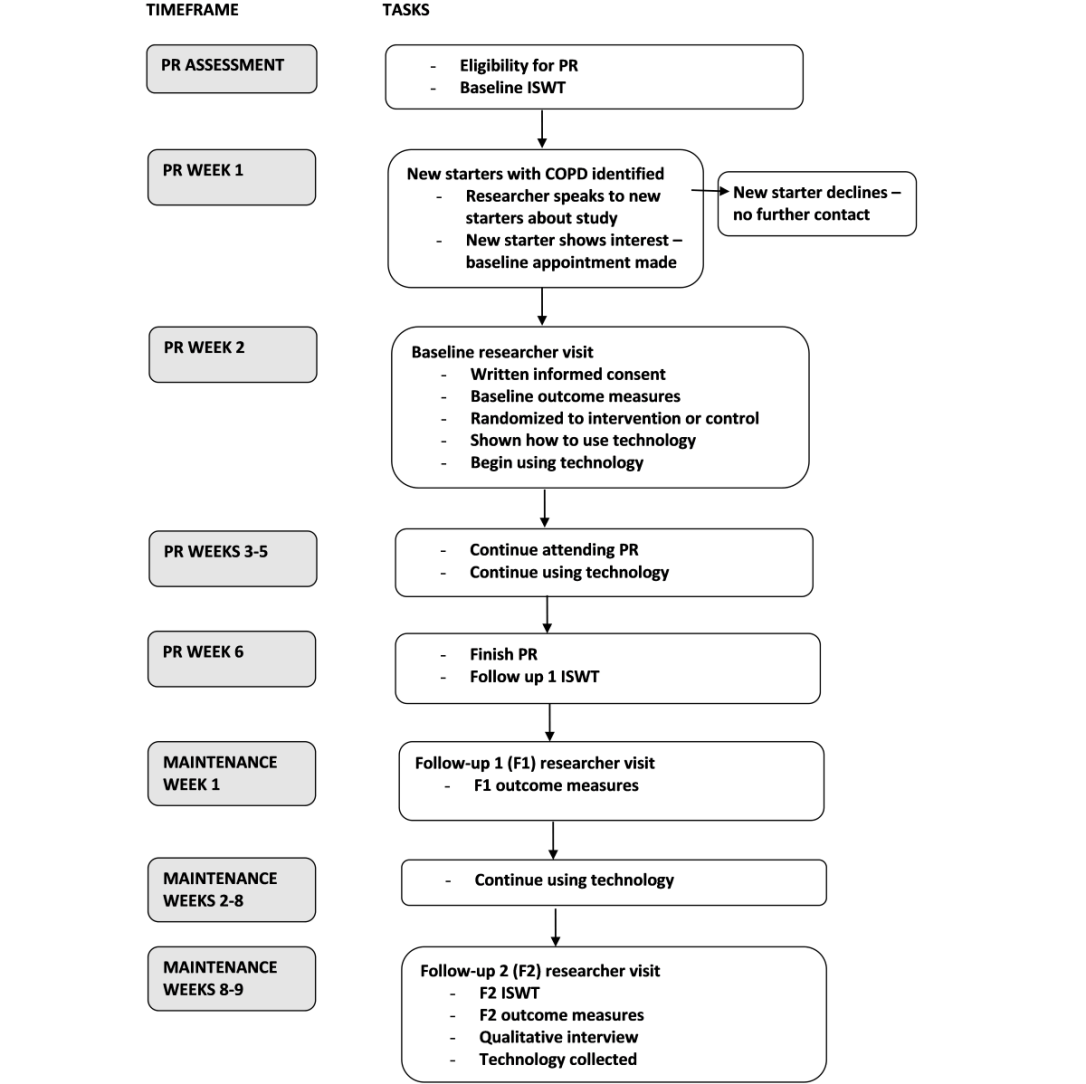
Summary of participants’ progress through the study. COPD: Chronic Obstructive Pulmonary Disease; ISWT: Incremental Shuttle Walk Test; PR: pulmonary rehabilitation.

Details of the study procedure are summarized in Figure 1. PR attendees were assessed for PR eligibility before starting the program. Participants attended PR twice weekly for 6 to 7 weeks. Each week PR physiotherapists informed the researchers if there were any new starters with COPD expected at PR sessions in the coming week. In their second PR session (during week 1), participants were asked if they would be happy for a researcher to speak with them about the feasibility study and what it would involve. Potential participants were given a participant information sheet and asked to contact the research team within the next week if they wished to take part.

### Data Collection and Randomization

If a participant agreed to take part, then a baseline appointment was organized with one of the research team members for week 2 of PR. At the baseline visit, written informed consent was obtained and the participant completed a set of questionnaires (see Quantitative Outcome Measures). The participant was then randomized to 1 of 2 conditions:

- Group 1 (intervention) used the app and activity tracker to monitor, maintain, and (if possible) increase their physical activity during their time in PR (the PR phase) and for a further 8 weeks afterwards (the maintenance phase).
- Group 2 (control) wore a *blinded* activity tracker for the PR phase and maintenance phase. A strong black tape was used to cover the activity tracker’s screen so the participant would not be able to see their step count. This group was also provided with a smartphone so that data from the activity tracker could automatically be sent to, and stored on, the phone.

Uneven randomization was used, whereby two-thirds of participants were assigned to group 1 and one-third to group 2. This was due to the need to assess the acceptability and usability of the intervention. A blinded researcher used sealed opaque envelopes to generate randomization. This method of allocation was chosen because it mimics the more rigorous software-generated randomization method used in RCTs, in a manner that was satisfactory for the purposes of the feasibility study.

After allocation, the researcher demonstrated the technology to the participant and set up baseline activity goals within the app. Participants were provided with an instruction manual and the research team’s contact details.

Participants then used the app and activity tracker, or wore a blinded activity tracker, during the PR phase in accordance with their allocation. An appointment was made for a member of the research team to visit the participant at the end of PR and take follow-up 1 (F1) measurements. At this point, the set of questionnaires applied at baseline was repeated.

Another appointment was made for 8 weeks after finishing PR (follow-up 2 or F2), ie, the end of maintenance. If possible, participants were seen at the PR facility so that a final incremental shuttle walk test (ISWT) could be conducted (see Quantitative Outcome Measures), a final set of questionnaires could be completed, and a semistructured qualitative interview could be conducted about their experiences on the study. The interview explored their experiences of using the technology, perceived benefits and barriers to using the technology, acceptability of randomization, perceived impact of the technology on physical activity, and tolerability of outcome measures (see Multimedia Appendix 1). During the final ISWT, participants wore a Fitbit Charge 2 (wrist-worn), a Fitbit One (hip-worn), and Axivity motion sensors on their hip and wrist to compare step count accuracy across different devices for individual participants. The devices were found to be comparable in accuracy, although detailed results of this comparison are not reported here. Finally, the research team retrieved the technology from the participant.

Clinical support (via the relevant PR team during PR sessions) and technical support (via the research team) was available to all participants throughout their time in the study. The protocol and all materials used for the study were reviewed by people with COPD for relevance and comprehensibility.

### Health Care Professionals

At the end of the recruitment period, PR team members (eg, respiratory physiotherapists) were contacted via email with a participant information sheet attached and were asked to contact the research team if they wished to take part in a qualitative semistructured interview or focus group discussion. The purpose was to explore HCPs’ opinions of using the technology alongside PR, including perceived benefits and barriers to using the technology, acceptability of the technology, and perceived impact on participants’ physical activity (see Multimedia Appendix 2).

### Quantitative Outcome Measures

The feasibility of using a number of quantitative outcome measures (for a future RCT) was assessed during the study, eg, for relevance and ease of completion. The System Usability Scale (SUS) [36] was also included as an objective measure of ease of use of the intervention. The quantitative outcome measures used are summarized in Table 2.

Exercise capacity was assessed using the ISWT, a well-validated measure [37] used as part of standard clinical practice by all three PR sites. Patients completed a baseline ISWT at their assessment visit and another ISWT in their final week of PR (ie, at F1) as part of standard practice. The research team requested these scores for all participants in the study and added an additional ISWT for participants at the end of the maintenance phase (F2). This was conducted at the PR facility by a respiratory physiotherapist. The research team collected all other outcome measures.

**Table 2:** Summary of measures taken from participants at different time points.

Measures		Baseline	Follow-up 1	Follow-up 2	Continuous
**Demographics**					
	Age, gender, ethnicity, postcode, medical conditions, previous PR^a^, and previous experience with technology	X^b^	N/A^c^	N/A	N/A
**Medical Research Council Breathlessness Scale**					
	COPD severity: 1 (“not troubled by breathlessness except on strenuous exercise”) to 5 (“too breathless to leave the house, or breathless when undressing”) [38]	X	N/A	N/A	N/A
**Physical activity: step count**					
	As measured by an activity tracker	N/A	N/A	N/A	X
**Physical activity: CHAMPS^d^ questionnaire**					
	CHAMPS [39]	X	X	X	N/A
**Exercise capacity: ISWT^e^**					
	ISWT [37]	X	X	X	N/A
**Functioning and quality of life: SGRQ^f^**					
	SGRQ [40]	X	X	X	N/A
**Anxiety and depression: PHQ-9^g^**					
	PHQ-9 [41]	X	X	X	N/A
**Exercise self-efficacy: Ex-SRES^h^**					
	Ex-SRES [42]	X	X	X	N/A
**Symptoms: CAT^i^**					
	CAT [43]	X	X	X	N/A
**Cost-effectiveness: EQ-5D-3L^j^**					
	EQ-5D-3L [44] for a future cost-effectiveness assessment	X	X	X	N/A
**Usability: SUS^k^**					
	SUS [36] to assess the usability of technology	N/A	X	X	N/A

^a^PR: pulmonary rehabilitation.

^b^X: Measure taken at indicated timepoint.

^c^N/A: not applicable.

^d^CHAMPS: Community Healthy Activities Model Program for Seniors.

^e^ISWT: incremental shuttle walk test.

^f^SGRQ: St George's Respiratory Questionnaire.

^g^PHQ: Patient Health Questionnaire.

^h^Ex-SRES: Exercise Self-Regulatory Efficacy Scale.

^i^CAT: Chronic Obstructive Pulmonary Disease Assessment Test.

^j^EQ-5D-3L: EuroQol 5 Dimensions 3 Level

^k^SUS: System Usability Scale.

### Quantitative Analysis

Descriptive statistics were calculated for demographic data, ISWT scores, and questionnaire-based outcome measures in SPSS. Data collected via the SMART-COPD app on amount and types of physical activity were summarized and explored in Microsoft Excel to provide insights into how the intervention was used.

### Qualitative Analysis

Qualitative interviews with patients with COPD and HCPs were transcribed verbatim, and a thematic analysis [45] was used to identify key themes within the data (using NVivo software). The first author explored the transcript data, taking notes on pre-existing and emerging themes. These notes were used to build a coding framework, which was used to code the transcript data within NVivo. All data within individual themes were then explored and summarized (including where there were differences between participants), to produce summaries of each theme and also to identify relationships between themes.

### Ethics

The study received NHS research ethics approval (15-YH-0458), as well as Health Research Authority and research governance approval from each NHS site. The feasibility study was registered on a clinical trials database (NCT02691104).

## Results

### Recruitment and Dropout Rates

A total of 30 people with COPD participated in the feasibility study: 19 participants were assigned to the intervention group and 11 were assigned to the control. 16 participants completed all three data collection points and 14 participants withdrew from the study. The groups were well matched on most demographics. Participants’ demographics are summarized in Table 3.

**Table 3:** Summary of participants’ baseline demographics and measurements.

Demographics		Intervention	Control	Overall
**Age (years)**				
	Median (IQR)	68.0 (63.0-72.0)	66.0 (60.0-70.0)	67.5 (60.0-70.5)
	Range	45-75	53-75	45-75
**Gender (frequency)**				
	Male	8	5	13
	Female	11	6	17
**Medical Research Council Breathlessness score^a^ (frequency)**				
	2	6	3	9
	3	3	3	6
	4	10	5	15
**PR^b^ attendances (frequency)**				
	First time	11	4	15
	Been before	8	7	15
**Ethnicity (frequency)**				
	White British	19	11	30
“**Regularly use computer” (frequency)**				
	Yes	8	6	14
	No	11	5	16
“**Regularly use mobile phone” (frequency)**				
	Yes	17	10	27
	No	2	1	3
“**Regularly use tablet” (frequency)**				
	Yes	8	7	15
	No	11	4	15

^a^1=least severe and 5=most severe.

^b^PR: pulmonary rehabilitation.

### Study Withdrawal

In the intervention group, 47% (9/19) of participants withdrew, and in the control group 46% (5/11) of participants withdrew; thus, attrition rates were similar for both groups. In total, 12 participants withdrew before F1: 3 participants withdrew either at baseline or within 2 weeks of baseline; and 9 participants withdrew within 3 to 6 weeks into the study. A further 2 participants withdrew between F1 and F2. More women withdrew compared with men (9/17, 53% vs 5/13, 38%, respectively). Withdrawers also had a lower median age compared with completers (median 65.5, IQR 59.8-70.5 years vs median 68.0, IQR 61.0-71.3 years, respectively). The most common (voluntarily given) reasons for withdrawing included ill health (n=5); withdrew from PR (n=4); burden of research, of technology, or of completing daily exercises (n=4); technical issues or frustrations with the technology (n=4); and disappointment at having been assigned to the control group (n=3). Some withdrawers cited more than one of these reasons. Several withdrawers (n=3) still liked the concept of the app, and 2 participants who had stopped attending PR would have liked to continue using the app.

Descriptive statistics on baseline outcome measures revealed a pattern of differences between participants who completed the study and those who withdrew. Withdrawers showed signs of having worse baseline disease severity and health compared with those who completed the study. These comparisons are summarized in Table 4.

Participants who withdrew had worse baseline scores on exercise capacity, quality of life, and depression compared with those who completed. However, sample sizes were small, so this finding should be interpreted with caution.

**Table 4:** Comparison of completed vs withdrawn participants on baseline outcome measures.

Baseline measures		Completed	Withdrawn
**Baseline ISWT score (meters)^a^**			
	Number	16	13
	Median (IQR)	255.0 (172.5-332.5)	170.0 (105.0-305.0)
	Range	90-550	40-490
**Baseline SGRQ score^b^**			
	Number	16	13
	Median (IQR)	52.0 (44.2-63.7)	62.4 (52.3-72.3)
	Range	32.3-78.1	42.3-79.6
**Baseline SGRQ current health question**			
	Number	16	14
	Very poor	0	3
	Poor	3	7
	Fair	10	3
	Good	2	1
	Very good	1	0
**Baseline CHAMPS score^c^**			
	Number	16	14
	Median (IQR)	2017.3 (1220.5-5237.3)	2468.5 (1193.8-3138.7)
	Range	68.0-9683.6	437.7-9644.3
**Baseline Ex-SRES score (%)^d^**			
	Number	16	14
	Median (IQR)	54.1 (27.7-72.0)	54.1 (32.4-69.7)
	Range	10.6-93.1	13.1-86.3
**Baseline PHQ-9 score^e^**			
	Number	16	14
	Median (IQR)	7.0 (4.3-12.5)	10.0 (5.5-15.0)
	Range	2.0-16.0	2.0-21.0
**Baseline CAT score^f^**			
	Number	16	14
	Median (IQR)	23.0 (16.5-25.0)	23.0 (16.8-26.8)
	Range	13.0-32.0	12.0-37.0
**EQ-5D score^g^**			
	Number	15	14
	Median (IQR)	8.0 (7.0-9.0)	8.0 (8.0-10.3)
	Range	5.0-11.0	6.0-12.0
**EQ-5D scale^h^**			
	Number	16	14
	Median (IQR)	60.0 (50.0-70.0)	50.0 (40.8-62.5)
	Range	28.0-90.0	20.0-90.0

^a^ISWT: incremental shuttle walk test; higher score=greater distance walked.

^b^SGRQ: St George’s Respiratory Questionnaire; lower score=better quality of life.

^c^CHAMPS: Community Healthy Activities Model Program for Seniors; higher score=more physical activity.

^d^Ex-SRES: Exercise Self-Regulatory Efficacy Scale; higher score=more exercise-related self-efficacy.

^e^PHQ-9: Patient Health Questionnaire for Depression; higher score=more depressed.

^f^CAT: Chronic Obstructive Pulmonary Disease Assessment Test; higher score=more COPD symptoms.

^g^EQ-5D-3L: EuroQol 5 Dimensions 3 Level score; higher score=worse health.

^h^EQ-5D-3L scale: EuroQol 5 Dimensions 3 Level scale; scored 0-100, where 100=best health they can imagine.

### Use of the Technology

For intervention participants, physical activity and usage data collected by the SMART-COPD app were transmitted by email using the smartphone’s mobile data connection. These data provided indications of how the app was used and how often. Table 5 summarizes the percentage of days on which each physical activity component of the app was used by each participant.

**Table 5:** Individual participants’ use of different features of the SMART-COPD app.

Participant	App use, no. days (%)	Steps recorded^a^, no. days (%)	Exercise recorded, no. days (%)	Daily walk recorded, no. days (%)	Total days participant had intervention, n
1^b^	12 (32)	15 (41)	6 (16)	9 (24)	37
2^b^	18 (64)	25 (89)	3 (11)	3 (11)	28
3	108 (90.0)	107 (89.2)	101 (84.2)	14 (11.7)	120
6	53 (51.0)	53 (51.0)	36 (34.6)	19 (18.3)	104
8	85 (98)	86 (99)	64 (74)	65 (75)	87
11^b^	23 (100)	18 (78)	1 (4)	10 (43)	23
14	85 (94)	83 (92)	57 (63)	79 (88)	90
15	62 (84)	66 (89)	1 (1)	56 (76)	74
19	100 (89.3)	99 (88.4)	41 (36.6)	41 (36.6)	112
21	19 (20)	79 (82)	8 (8)	0 (0)	96
22^b^	69 (100)	68 (99)	4 (6)	58 (84)	69
23^b^	6 (100)	6 (100)	3 (50)	5 (83)	6
24	19 (17)	31 (28)	5 (5)	15 (14)	111
27	71 (63)	88 (79)	7 (6)	20 (18)	112
30	94 (96)	13 (13)	5 (5)	83 (85)	98
Mean	55 (71)	56 (72)	23 (29)	32 (41)	78

^a^Steps were recorded by an activity tracker even if the Self-Management supported by Assistive, Rehabilitative, and Telehealth technologies-Chronic Obstructive Pulmonary Disease app itself was not used.

^b^Participants who later withdrew from the study.

A total of 3 intervention participants withdrew immediately or within 1 to 2 weeks of receiving the technology. No app use data were recorded for these participants. One intervention participant (17) withdrew 5 weeks into the study but had no app use data recorded. Technical issues were noted for this participant (eg, the activity tracker not holding its charge); however, it is unclear if this was the reason for the lack of data from this participant.

On average, the SMART-COPD app was used on 73% of days on which it was deployed to a participant, although individual participants’ usage patterns varied widely. The steps component of the intervention was the most frequently used physical activity strategy overall. However, use of the steps component decreased for some participants when they moved into maintenance. Participants generally maintained consistent (high or low) usage levels for daily walks and exercises across the PR and maintenance phases.

### Outcome Measure: Step Counts

Participants did not show a consistent pattern overall with respect to whether their step counts increased, decreased, or stayed the same over time. Most intervention participants who completed the study had a near-complete dataset for the full study timeframe. Only 2 control participants had near-complete datasets. Some gaps in the data were explained by technical issues or by participant illness, but many gaps were unexplained.

### Outcome Measure: Incremental Shuttle Walk Test

The ISWT was found to be relevant and appropriate as an outcome measure for exercise capacity, as this was routinely collected in the three PR sites and participants were used to completing it. However, the logistics of carrying out a third ISWT at F2 (when participants had already been discharged from the PR service) at times proved challenging owing to the need to coordinate availability between participants, the research team, and the PR team.

### Outcome Measure: Questionnaires

All participants were generally satisfied with the questionnaires and length of time needed to complete them. Some participants requested help from the researcher to complete them, eg, asking the researcher to read questions aloud and complete answers on their behalf. Some questions felt repetitive to participants if a similar question was included on more than one questionnaire. However, the only questionnaire that caused significant problems in completion was the Community Healthy Activities Model Program for Seniors (CHAMPS) self-reported physical activity questionnaire. Participants did not identify with the *Americanized* nature of included activities or the wording of some of the questions (eg, *shooting pool*), which were felt to be less relevant to a British population. Researchers also noted problems in participants’ understanding of the timeframe to which the questions applied and confusion over calculating how often, or for how long, each activity was completed.

### System Usability Scale

Individual participants’ SUS scores at F1 and F2 are summarized in Table 6 (for intervention participants who completed the study). Scores are out of 100, with a higher score indicating greater usability.

SUS scores were generally high compared with the industry standard average score of 68 [42], indicating the intervention had a *higher than average* usability level across technological systems from multiple industries. Participants’ SUS scores generally (but not always) increased with time spent using the technology.

**Table 6:** Individual intervention participants’ System Usability Scale scores at Follow-up 1 and Follow-up 2.

Participant	Follow-up 1	Follow-up 2
3	97.5	90
6	80	92.5
8	85	90
14	90	90
15	75	85
19	77.5	82.5
21	65	5
24	55	55
27	67.5	97.5
30	Missing	Missing

### Qualitative Results

#### People With Chronic Obstructive Pulmonary Disease: Reactions to the Intervention

The interviews captured the views of 16 participants who completed the study (n=10 intervention group; n=6 control group). Qualitative data relating to the intervention were categorized into six main themes: technology; technical issues; previous experience (with digital technology); integration with PR; control group issues; and involvement in the project.

#### Technology

The intervention was generally well liked and well accepted among participants who completed the study. Most of these participants found the technology easy to use and were able to incorporate it into their daily routine, eg, putting on the activity tracker in the morning:

Yes, absolutely no problem, once I’d got, had a shower and you know put it on and just carried on what I was doing… even forgetting sometimes I’d got it on.

Patient 28

Around half of (completed) the participants felt they had benefitted (physically or psychologically) through enhanced confidence, monitoring of physical activity, and incentivization to exercise:

If I hadn’t found this technology erm I would probably be just sat at home watching TV… it would never occur to me to do exercise at home and to move.

Patient 3

Many participants found the technology motivational. Feedback on activity and goal attainment motivated them to do more:

You look at it in the day and... see how many steps you’ve done and it encourages you I think.

Patient 6

Two participants did not use the intervention in this way—they were already active and used the intervention to monitor activity that they were already doing rather than trying to increase it:

It’s just the way I live. I’ve not done anything different to what I normally do… made me realise how much I was doing, or how little I was doing but I wouldn’t say it increased what I put into it.

Patient 15

One participant (21) disliked the smartphone and SMART-COPD app and found them difficult to use (supported by her F2 SUS score), although she was happy to continue wearing the activity tracker:

Rubbish cause I couldn’t do it, couldn’t do it, no way could I do it… I’ve never been able to use one of them phones for a start.

Patient 21

Participants rarely updated goals. Reasons included conforming to what the *experts* (physiotherapists or researchers) recommended (and the physiotherapists did not explicitly tell them to increase these goals) and keeping the goal achievable:

I didn’t want to do it too much because I knew I’d be disappointed if I didn’t reach the goal I’d set.

Patient 3

Opinions of the daily walk and exercise sections of the app were mixed. Even where the exercise section was used, the videos were not well liked—they were viewed as repetitive or participants were put off by people in the videos they perceived as less able than themselves:

I haven’t used the exercise bit because I looked at the exercise thing and it’s all people sat in chairs and looks like they’re all in old people’s homes, I’m a little bit more active than that.

Patient 15

Reasons for low use of the daily walk section included difficulty getting the smartphone to register the walk and not wanting to walk outside alone:

I don’t go for daily walks so I thought I’ll give it a try in the house and I had to walk at such a speed [for the walk to register via the accelerometers in the smartphone] I was frightened of banging into doors and all sorts so in the end I decided not to do the daily walks.

Patient 3

There were misunderstandings around the daily walk section of the app, with a few participants referring to the step count and daily walk sections interchangeably. At least two participants did not understand that the phone needed to register continuous movement and hence needed to be placed in the hand or the pocket to record the duration of the walk:

I can have walked for twenty minutes at the rehab centre on the err treadmill and it doesn’t show it, it never works… I have on the machine you know I stand it up on the machine.

Patient 6

Many participants, from both groups, discovered the Fitbit app on the smartphone, even though it was not intended that either group should use it directly. Installation of the app was necessary for data synchronization but could be discovered by participants with an experience of, or curiosity about, using smartphones. Several used this, rather than the SMART-COPD app, to monitor their step counts. At least two control participants used the Fitbit app to monitor their steps and increase their activity despite it being against the planned intervention:

Just at odd times I did [check steps on Fitbit app], yes, yes just odd times, I didn’t cheat at all.

Patient 28, control

Overall, the activity tracker was the most liked component of the intervention, and a few participants expressed a preference for using the activity tracker alone and leaving out the SMART-COPD app:

With Fitbit bit, chuck phone away and just leave Fitbit. As long as you’ve got the app that you can have on your own phone.

Patient 24

Participants who already owned smartphones often expressed a preference for having the SMART-COPD app installed on their own phone rather than carrying and charging extra equipment. In total, 3 participants were conscious of not wanting to damage or lose the study’s equipment, which would be less of a worry if they were using their own technology:

The only thing that did affect me was the fear of losing it all… it’s using somebody else’s equipment and being responsible for it.

Patient 6

Most participants did not carry the smartphone with them when they left the house. Participants were also less likely to use the technology if they were unwell, busy, or on holiday:

I just want to get up and go. If I’m taking my dog out for a walk I’ve got enough trouble getting leads, making sure there’s bags on it and harness on dog and treats to make sure they come back without having to take [the phone].

Patient 24

#### Technical Issues

The Fitbit device frequently stopped synchronizing with the Fitbit app. This happened for at least six participants. These occurrences were sometimes due to the device not being charged and, in one case, a participant accidentally deleting the Fitbit app from the smartphone. However, in most cases, these communication errors were unexplained:

They weren’t getting no data through and I said well I don’t know whether Fitbit what’s not charging or phone what’s not charging.

Patient 25

There were a number of reports of itchiness, rash, or discomfort from wearing the activity tracker, particularly in warm weather:

I bruise very easily, my skin is wafer thin and it was very uncomfortable and it marked both arms so it had to go.

Patient 3

A few participants who experienced early problems were offered the hip-worn Fitbit One as an alternative and seemed to get on well with this option. A few other participants expressed a preference for a hip-worn activity tracker at the interview stage, eg, for discretion.

There were at least three cases of the activity tracker catching on clothes and falling off:

I lost it in the churchyard once; luckily it was still there when I went back... it had caught on my sleeve and just pulled off.

Patient 15

#### Previous Experience

Many participants had previous experience with using digital technology, such as smartphones and computers, and a few had a prior interest in technology:

[Talking about the game Pokémon Go] So I quite enjoy that and I’ve starting using my eggs as distances on how much I’ve walked.

Patient 24

However, some participants had little or no previous experience, and these participants were more likely to encounter difficulties with using the intervention:

I don’t know how they work and I’m not interested to be honest. The learning how to work it, in my opinion I’m at wrong time, wrong side of life now to start worrying about technology.

Patient 25

No one had previously used a Fitbit. Some participants knew about Fitbit devices through advertisements or family members, but had perceived them as being irrelevant for themselves or as being *only for athletes*:

I wouldn’t have known about that Fitbit cos I weren’t interested in things like that… I never took no notice. I always thought people did it when they went in gym, you know like they bought a Fitbit just to cycle and things like that.

Patient 29

Some participants felt the intervention was less suited to people with COPD who were older, less technologically *savvy*, with more severe disease, or living alone, despite the app being perceived as easy to use:

I could understand how some people that are a bit older than me would struggle with it if they hadn’t got any support in the house. I mean I’ve got a husband and he’s pretty good with technical things but I didn’t have to really ask him, I figured it myself.

Patient 6

#### Integration With Pulmonary Rehabilitation

There were few reported issues with the intervention being used alongside PR, and many participants liked their concurrent use. Most participants did not speak to the PR team about the technology. However, where they did speak to physiotherapists, they often found the HCP did not know much about the technology or the study (though they still showed an interest and tried to help with any issues):

They didn’t know how it worked. They didn’t, you know because I did ask at the beginning I was a bit flummoxed with it all erm and I did ask the physio that was there then and she, she had a look but she couldn’t tell me, but I figured it out myself in the end.

Patient 6

#### Control Group

Most control group participants would have liked to see and use the intervention but were usually happy to take part. A few intervention participants felt they would have been disappointed to receive the control condition but stated they would have continued with the study:

Well, I honestly felt a bit disappointed... because erm I did want to experience trialling erm the Fitband [sic] as it should be used... but I realise the importance of that um, you need both aspects of it, so, I was still happy to do it.

Patient 07, control

One control group participant who had no prior experience with digital technology stated he would have withdrawn if he had been allocated to the intervention group:

I’d have probably put it back in box and have rung em back up and said I don’t want to do it, it’s too much for me this, I’m not into it.

Patient 25

When technical issues occurred, control group participants were less likely to realize that there was a technical issue compared with intervention group participants, as they could not see the Fitbit display or the Fitbit app (unless they had *discovered* it). When participants did realize that there was a technical issue, it was usually because they were using the Fitbit app or noticed the activity tracker no longer had a flashing light underneath:

Well part of it was when it wasn’t working, which I didn’t know, when I was unaware of that.

Patient 28

One control group participant had been given a Fitbit Charge 2, whose charging cradle differs from the Charge HR. For this participant, the black tape covering the screen interfered with the charging cradle, which caused weeks of problems getting data from this participant (until the root cause was discovered).

#### Involvement in the Project

Participants did not generally contact the research team if they were having problems using the technology or were experiencing technical issues. These problems were usually detected when members of the research team called participants to arrange data collection visits. Some participants had lost the research team’s contact details; others seemed reluctant to *bother* researchers with issues they feared were due to their own *incompetence* or inexperience with technology:

No I’ve never contacted you. I was thinking about it in the beginning because I wasn’t getting, as I say I was a bit flummoxed by it all, but I figured it out so I didn’t, I’ve not phoned up or anything, I’ve not had any contact other than the visits.

Patient 6

One participant who was initially resistant to using technology decided to try it after hearing the researcher’s explanation of the study:

Well when you when they first asked me, would I, you know at pulmonary rehab, would I take one and I was thinking no I can’t be doing with that, you know. Can’t be doing with that… because I didn’t understand it… until it was explained and then I thought, yes I’m going to have that, yes.

Patient 14

Participants enjoyed taking part in the study and were mostly happy with their contact with the research team. Some participants had purchased, or planned to purchase, their own Fitbit to continue using after the study had finished:

It would never have entered my head to go and buy something to improve my condition, never, erm but now I’ve got another Fitbit waiting for me when I go home.

Patient 03

#### Health Care Professionals: Reactions to the Technology

A total of 5 HCPs took part in either a focus group discussion or an interview exploring their experiences of participating in the study. Five themes relevant to HCPs’ experiences with the technology are discussed: technology; recruitment; communication; workload; and suggested improvements.

##### Technology

One physiotherapist (with personal experience of using activity trackers) described the SMART-COPD app as *clunky* and visually unattractive. The staff felt some participants were motivated by the technology to achieve physical activity goals between PR sessions, although they also noted that some participants are naturally more motivated regardless of whether they have technology.

##### Recruitment

The staff reported that some participants *got on* with the technology better than others, and that it was usually (but not always) younger and more technologically experienced participants who adapted quickly and gained the most benefit. However, 2 staff members pointed out that older people are becoming more technologically experienced as the years progress, so this may not be an issue for future generations.

The study did not have any inclusion criteria around previous experience with technology. However, there were hints that a small number of potential participants may not have been put forward to the research team if staff members felt they would not benefit from the intervention, eg, if they had never used digital technology or did not seem motivated to benefit from PR. The staff also heard a few control group participants expressing disappointment with their allocation, eg, wondering about the purpose of their involvement.

##### Communication

During the feasibility study preparation, the research team conducted workshops with PR staff and involved them in planning the logistics of the intervention and the study. Unfortunately, it was (understandably) difficult to speak with the entire PR team; therefore, not all staff were briefed in-person on the study and technology, and key information did not always filter through to the entire team. In addition, physiotherapists within PR teams are frequently rotated to different locations. In the time it took to complete development work and get ethical amendments approved, some PR team members had changed, and even those who were involved in earlier stages of the study did not always recall how the technology or the study worked.

This was reflected in the staff interviews, in which physiotherapists often reported that they did not know much about the technology or the wider study. Owing to their own experiences with technology, PR team members were more able to help with generic smartphone issues but were not usually experienced with using activity trackers or the SMART-COPD app.

##### Workload

The study did not have a large impact on workload, as PR physiotherapists are accustomed to speaking with individual patients during PR sessions and checking their progress. One difficulty, however, was that the research team was not always told in good time when a new starter would be attending a PR session, especially when this was decided at short notice or when this coincided with staff absence on either side. Physiotherapists sometimes reported difficulties deciding appropriate physical activity goals for participants, as they did not think in terms of the number of daily steps a person with COPD should aim to achieve. There were also difficulties organizing appointments for conducting F2 ISWT tests (which were not a part of normal service delivery). Overall, it was felt that the SMART-COPD intervention had become a tool used alongside PR rather than incorporated within PR, although it was thought in some cases to have enhanced people’s PR experience and the benefits they gained from it.

##### Suggested Improvements

Suggestions for improving the study included a dedicated central email list to improve communication and sharing of responsibility for appointments and tasks. One HCP suggested asking participants to wear a *blinded* activity tracker for a week before starting the study to gain a better baseline physical activity level. This would help with setting appropriate physical activity goals for the individual and would help with working out whether the intervention was effective for that individual.

## Discussion

### Principal Findings

This randomized feasibility study examined the feasibility and acceptability of using a wearable activity tracker and the SMART-COPD app both within and following PR to encourage people with COPD to increase, or at least maintain, their physical activity levels (RQ1). The study also explored the feasibility of conducting a future RCT to investigate the effectiveness of the intervention (RQ2). The intervention shows potential in helping a subset of people with COPD to achieve physical activity goals. However, both the intervention and methods used would need to be modified if a future RCT were to be conducted.

### Acceptability of the Intervention

A total of 30 people with COPD were recruited to the study. The 16 participants who completed the study were generally positive about the intervention (or liked the concept if they were in the control group). Some believed they had gained tangible benefits from using the intervention, both in terms of motivation to achieve physical activity goals and subsequent benefits to their physical or psychological health. Most participants who completed the study found the technology easy to use and most experienced no problems incorporating it into their daily lives.

However, almost half of the participants (n=14) withdrew from the study. Some participants seemed to find the prospect of using digital technology for physical activity monitoring to be daunting or overwhelming. This was supported by the reasons given for withdrawing, and qualitative feedback from both patients and staff, which indicated that people who were older or less experienced with digital technology or did not have support at home might be wary of using this type of intervention. Overall, people who withdrew from the study had worse baseline scores on exercise capacity, quality of life, and depression compared with those who completed the study, which indicates that people with better COPD-related health may gain more benefits from mHealth-based interventions. This claim must however be interpreted with caution due to the small sample size.

Although mHealth is a promising intervention for the self-management of COPD, the evidence base around mHealth is currently mixed and underdeveloped [46-48]. In this study, there was arguably a dichotomy between people who completed the study and gained perceived physical and psychological benefits from the intervention and those who did not easily adopt the technology or found it to be a burden. This has been found for related interventions such as telehealth [46,49,50] and supports McCabe et al’s [19] hypothesis that patients with COPD with greater interest in technology may gain greater benefits from mHealth interventions. In addition, a recent literature review of wearable technologies for physical activity in COPD identified only a small number of RCTs with highly heterogeneous technologies and study designs, meaning no conclusions could be drawn about their effectiveness [51]. More evidence is needed on the use of wearables in COPD, along with an improved ability for accurate step count detection, and more robust guidelines are needed for clinical staff to implement wearable technologies for COPD [51].

The SMART-COPD app itself mostly worked well. However, one of the most challenging technical issues was the failure of activity data to be transmitted from the activity tracker to its corresponding smartphone app. This was frustrating for all concerned as it meant some participants’ physical activity data were not recorded, and indeed technical issues were cited by several participants as a reason for withdrawing. The wrist-strap also caused discomfort and could catch on clothes and fall off. These issues would need to be resolved if the intervention were to be used on a wider scale.

Although the intervention seemed acceptable as a tool for use alongside standard PR, a full integration of the intervention within service delivery was problematic. Communication errors occurred between the research team and PR team despite the best efforts of everyone involved. In addition, the logistical difficulties of briefing entire PR teams, coupled with the tendency of PR physiotherapists to be moved between teams, resulted in some PR team members feeling uninformed about the technology and the wider study. However, PR staff were still supportive of the intervention, especially when participants within their service told them about their positive experiences.

There are potential patient-level advantages to incorporating the SMART-COPD intervention within PR, eg, access to clinical support when first using the app. However, it is worth noting that a large proportion of patients with COPD referred to PR do not attend or do not complete the course [52,53]. Reasons for nonattendance and noncompletion include perceived lack of benefit, disruption to usual routine, poor access to transport, greater disease severity, lower quality of life, and greater symptoms of depression [53,54]. Even if the intervention were deployed within PR in the future, a large number of people with COPD would not have the opportunity to use this method of self-managing their physical activity. Therefore, future research should explore other ways of delivering mHealth interventions to people with COPD who do not access PR.

### Feasibility of the Study Design

Almost half of recruited participants dropped out of the study. This finding has implications both for the acceptability of the intervention and for the sample size of a future RCT, which would need to account for high dropout rates. It could be argued that the inclusion criteria should be modified to target people with COPD who are deemed more likely to engage with this type of intervention. However, 1 or 2 individuals readily adopted the technology against their own or the physiotherapists’ expectations. HCPs in this study made the point that future generations coming through PR are likely to have more experience with digital technology and might therefore be more likely to engage with mHealth. These points suggest that the inclusion criteria should not exclude potential participants based on age, (actual or perceived) COPD-related health, or (actual or perceived) aptitude toward, or previous experience with, technology.

Most outcome measures tested were found to be suitable for use in a future RCT. The ISWT is routinely conducted in PR and would be suitable as an outcome measure for exercise capacity in a future trial. However, in this study, we were unable to identify a suitable outcome measure for physical activity. Fitbit One and Fitbit Charge were both accurate in counting participants’ steps during the F2 ISWT (when compared with Axivity sensor readings). However, control participants were more likely to have gaps in their step count data, and so these data are unlikely to have constituted an adequate comparison if this was an RCT. In addition, although control group participants could not see their step count on the Fitbit screen or on the smartphone’s home screen, some control group participants discovered the Fitbit app on the smartphone (a necessity for recording step count data) and were able to see their step counts. This meant that some control group participants were monitoring their step counts despite the *blinded* activity tracker. Thus, it would currently be challenging to use step count as a between-group comparative outcome measure in any future study. Participants also experienced difficulties completing the CHAMPS questionnaire for physical activity in older adults, thus ruling out this option as an outcome measure for physical activity. One physiotherapist suggested using a blinded activity tracker to take a baseline step count for participants between their assessment visit and beginning PR, which could provide a more reliable indication of physical activity changes within individual participants rather than between experimental groups in an RCT.

Some (though not all) participants were disappointed to be assigned to the control group, and in some cases, it was difficult for participants to appreciate the purpose of the control condition. For a future study, this may be resolved by giving all control group participants the opportunity to try using a wearable activity tracker at some stage in the study, eg, after data collection.

### Implications for Future Development of the Intervention

Smartphones and wearables are two technologies predicted to transform health care provision in the coming decades [21]. Participants who helped codevelop the SMART-COPD intervention stressed the importance of being able to personalize the intervention. This led to the inclusion of three different strategies to encourage maintenance of physical activity. However, when using the intervention in the *real world*, this approach proved too complicated. The daily walk and exercise components of the app were not widely used, and in some cases proved confusing for participants and PR team members alike. The more complex a technological intervention is, the less usable it is likely to be [26], and problems with usability may negatively impact participants’ motivation to continue with the intervention or the behavior [18]. Although efforts were made to design the intervention based on the needs and capabilities of people with COPD, more could have been done to achieve a truly co-designed intervention [55]. In addition, the self-selected participants who helped codevelop the SMART-COPD intervention were mostly experienced with digital technology and may not have been fully representative of the general COPD population.

Our results also suggest that people’s engagement with technology might wane over time (as had happened with some of our participants), although this study does not provide any indication as to whether participants began disengaging from the technology only or from the entire health behavior. This pattern was not present for all participants and the use of different components of the intervention differed between participants, which implies there were individual differences in how participants interacted with, and responded to, the intervention.

This study shows that participants also need the capability [18] to learn how to use the technology. People with less experience of digital technologies may have had less capability to use the intervention, and thus were more likely to leave the study. Participants appeared to have differing experiences of the SMART-COPD intervention depending on their previous experience with digital technology and their baseline health. This finding is indicative of the need to consider the circumstances, motivations, and capabilities of individual participants. The SMART-COPD intervention was complex, and the COPD population is complex and has complex needs. One intervention does not fit all, and future investigations of similar mHealth-based interventions need to consider individual factors as well as group factors when determining who could gain the most benefit [19].

Our results suggest that a future version of the app would need to be simplified. One option would be to adopt an existing commercially available activity tracker (eg, Fitbit) and its associated app to monitor step count and the completion of individually relevant step count goals. This approach has the benefit of not needing to be updated or maintained directly by the (resource-limited) research team, instead relying on a technology that is widely available and has a commercial team developing and maintaining it. However, while the SMART-COPD app was specifically designed to consider the needs of users with COPD and included simplification of the presentation of step count information collected via the activity tracker, commercially available apps are not designed for this population. Hence, we would need to determine whether patients with COPD without prior experience with digital technology could benefit from this approach (and, indeed, to ensure that the motivational aspects of commercial apps are not harmful or counter-productive for this population).

### Strengths and Limitations

One strength of the feasibility study was that the SMART-COPD intervention was tested with people with COPD in a real-life setting, with real-life complexities and challenges, and over a period of several months. The study involved both people with COPD and relevant HCPs and tested out design elements of an RCT. In addition, to our knowledge, no one has previously used a wearable activity tracker to monitor step count for both an experimental and a control group.

Potential limitations of the study include technical issues affecting participants’ experiences of using the intervention, and the effect of those issues on data completeness. The study also experienced a high dropout rate. While this in itself provided valuable information on the usability and acceptability of the intervention, it also affected data completeness. We were unable to formally interview these participants to fully understand their experiences with the technology and their reasons for leaving the study.

The resource-limited nature of the feasibility study meant we were only able to include three PR sites: these sites may not have been representative of PR services (and COPD populations) across the United Kingdom. To practically conduct the study, we also had to use more than one researcher for recruitment and data collection. Researchers were trained on the use of the technology and on how to introduce both the study and the technology to participants. However, there may still have been differences in how each researcher introduced and explained the technology and the study, which may have influenced some participants’ interactions with the technology.

However, all of the above strengths and limitation issues reflect the wider complexities of assessing mHealth interventions in real-world settings.

### Conclusions

Overall, the SMART-COPD intervention was well liked and perceived as easy to use and easy to incorporate into participants’ daily lives by those who completed the study. However, there was a high dropout rate which implies high rates of people who were eligible for the intervention but who did not easily adopt the technology, or else disliked the study design (eg, because of allocation). The data suggest that people with COPD who had worse baseline health were more likely to withdraw from the study, which may indicate that this patient group is harder to reach with mHealth interventions. The results suggest the intervention would need to be simplified for future use, eg, by focusing on step count only, with the possible sole use of a wearable activity tracker and an associated app. This finding contradicts a key finding from our earlier codevelopment work, which emphasized the importance of having a multi-option personalizable intervention. In a future RCT, the control group would be offered an opportunity to use the intervention and either the ISWT or a within-subject measure of step count should be considered as a primary outcome measure. Any future evaluation of the intervention would need to consider individual factors that affect the usability, acceptability, and efficacy of the intervention.

## Appendix

Multimedia Appendix 1 Chronic Obstructive Pulmonary Disease Patient Interview Guide.

Multimedia Appendix 2 Healthcare professional interview guide.

Multimedia Appendix 3 Screen Flow for SMART COPD App.

Multimedia Appendix 4 CONSORT-eHealth Checklist (V 1.6.1).

## Abbreviations

BCW: Behavior Change Wheel
CC: chronic condition
CHAMPS: Community Healthy Activities Model Program for Seniors
COPD: Chronic Obstructive Pulmonary Disease
HCP: health care professional
ISWT: Incremental Shuttle Walk Test
mHealth: mobile health
NHS: National Health Service
NIHR: National Institute for Health Research
PR: pulmonary rehabilitation
RCT: randomized controlled trial
SMART: Self-Management supported by Assistive, Rehabilitative, and Telehealth technologies
SUS: System Usability Scale
