# Supplementary material for: The Use of a Smartphone App and an Activity Tracker to Promote Physical Activity in the Management of Chronic Obstructive Pulmonary Disease: Randomized Controlled Feasibility Study
Source: JMIR Mhealth Uhealth. 2020 Jun 3;8(6):e16203. doi: 10.2196/16203 (PMC7301262; doi:10.2196/16203)
Supplement: Multimedia Appendix 1 [file mhealth_v8i6e16203_app1.docx]

[ENSURE INFORMED CONSENT HAS BEEN OBTAINED AND THE RECORDER IS SWITCHED ON]

[MAY NEED TO MODIFY WORDING OF QUESTIONS DEPENDING ON CIRCUMSTANCES AND WHICH GROUP THE PARTICIPANT WAS IN]

[PLEASE NOTE THIS DOCUMENT IS A GUIDE ONLY: WORDING AND QUESTION ORDER / COVERAGE ARE FLEXIBLE DEPENDING ON THE PARTICIPANT]

**First of all, how would you say you have found the experience of using the technology?**

**Is there anything you have liked about using the technology?**

PROMPTS:

- Ease of use
- Design
- Convenience
- etc

**Is there anything you have disliked about it?**

PROMPTS:

- Ease of use
- Interference in daily routine
- Technical issues
- Remembering to wear
- Not wanting to be in control group
- etc

**In your opinion, did using the technology help you to increase or maintain your physical activity? Why / why not?**

**In your opinion, do you feel that you would use this technology long term if it was available to you?**

**What are your views on using the app in combination with Pulmonary Rehabilitation?**

PROMPTS:

- Support
- Length of support
- etc

**How have you found your involvement in the overall project?**

**Did you need to contact the research team during the project?**

PROMPTS:

- What about?
- Helpful or not?

**Did you need to speak to the pulmonary rehabilitation team (about the technology) during the project?**

PROMPTS:

- What about?
- Helpful or not?

**Did you need to use any health services other than pulmonary rehabilitation during your time on the project? (e.g. GP, hospital, others)**

**Did anything happen in your life which made it more difficult to use the technology? (e.g. holiday, hospitalisation etc)**

**Are any changes needed to the technology?**

**What are your views on the fact that you could be randomly assigned to receive the app or not receive the app?**

**Were there any specific tests or questionnaires which you felt were particularly relevant or not relevant?**

PROMPTS:

- Time-consuming
- Difficult to understand
- etc

**Do you have any final thoughts or comments which you would like to add?**

**Thank you very much for your time!**
