# Supplementary material for: The Use of a Smartphone App and an Activity Tracker to Promote Physical Activity in the Management of Chronic Obstructive Pulmonary Disease: Randomized Controlled Feasibility Study
Source: JMIR Mhealth Uhealth. 2020 Jun 3;8(6):e16203. doi: 10.2196/16203 (PMC7301262; doi:10.2196/16203)
Supplement: Multimedia Appendix 2 [file mhealth_v8i6e16203_app2.docx]

[ENSURE INFORMED CONSENT HAS BEEN OBTAINED AND THE RECORDER IS SWITCHED ON]

[MAY NEED TO MODIFY WORDING OF QUESTIONS DEPENDING ON THE PARTICIPANT’S ROLE]

[PLEASE NOTE THIS DOCUMENT IS A GUIDE ONLY: WORDING AND QUESTION ORDER / COVERAGE ARE FLEXIBLE DEPENDING ON THE PARTICIPANT]

**First of all, how would you say you have found the experience of using the technology within Pulmonary Rehabilitation (PR)?**

**Is there anything you have liked about using the technology?**

PROMPTS:

- Ease of use
- Fit with PR
- etc

**Is there anything you have disliked about it?**

PROMPTS:

- Ease of use
- Interference in PR routine
- Technical issues
- etc

**In your opinion, did using the technology help patients to increase or maintain their physical activity? Why / why not?**

**In your opinion, were patients accepting of the idea of using technology within PR? Why / why not?**

PROMPTS:

- Were there any ‘types’ of patients who were more, or less, interested in the idea?

**What are your views on using the app in combination with PR?**

PROMPTS:

- Support
- Length of support
- etc

**How have you found your involvement in the overall project?**

**Would any changes be needed to the current care pathway to continue using the technology within PR?**

**Are any changes needed to the technology itself?**

**Were there any specific tests or questionnaires which you did which you felt were particularly relevant or not relevant?**

PROMPTS:

- Time-consuming
- Difficult to understand
- etc

**Do you have any final thoughts or comments which you would like to add?**

**Thank you very much for your time!**
