# Supplementary material for: The Use of a Smartphone App and an Activity Tracker to Promote Physical Activity in the Management of Chronic Obstructive Pulmonary Disease: Randomized Controlled Feasibility Study
Source: JMIR Mhealth Uhealth. 2020 Jun 3;8(6):e16203. doi: 10.2196/16203 (PMC7301262; doi:10.2196/16203)
Supplement: Multimedia Appendix 3 [file mhealth_v8i6e16203_app3.pptx]

## Slide 1
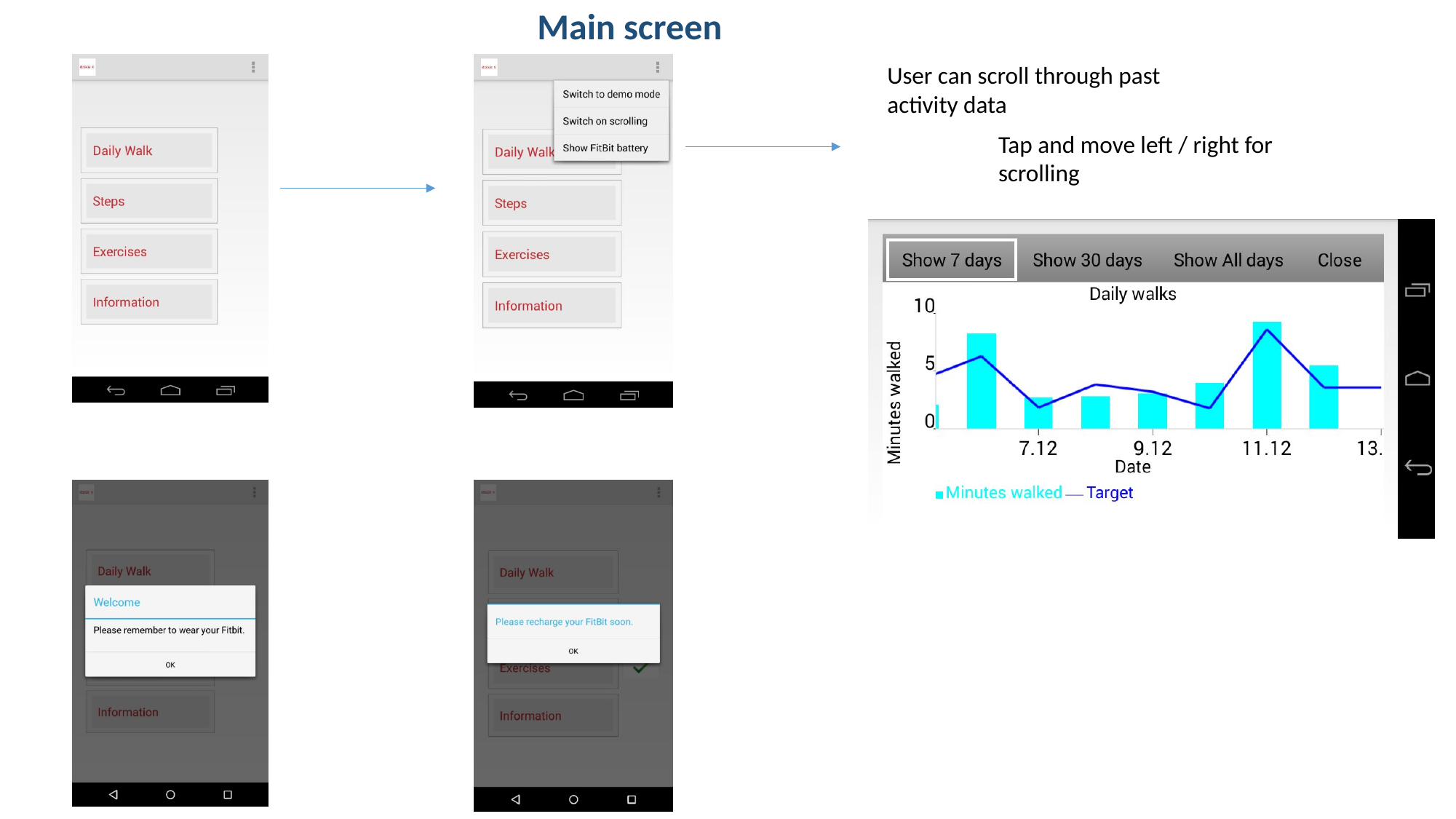

Main screen
User can scroll through past activity data
Tap and move left / right for scrolling

## Slide 2
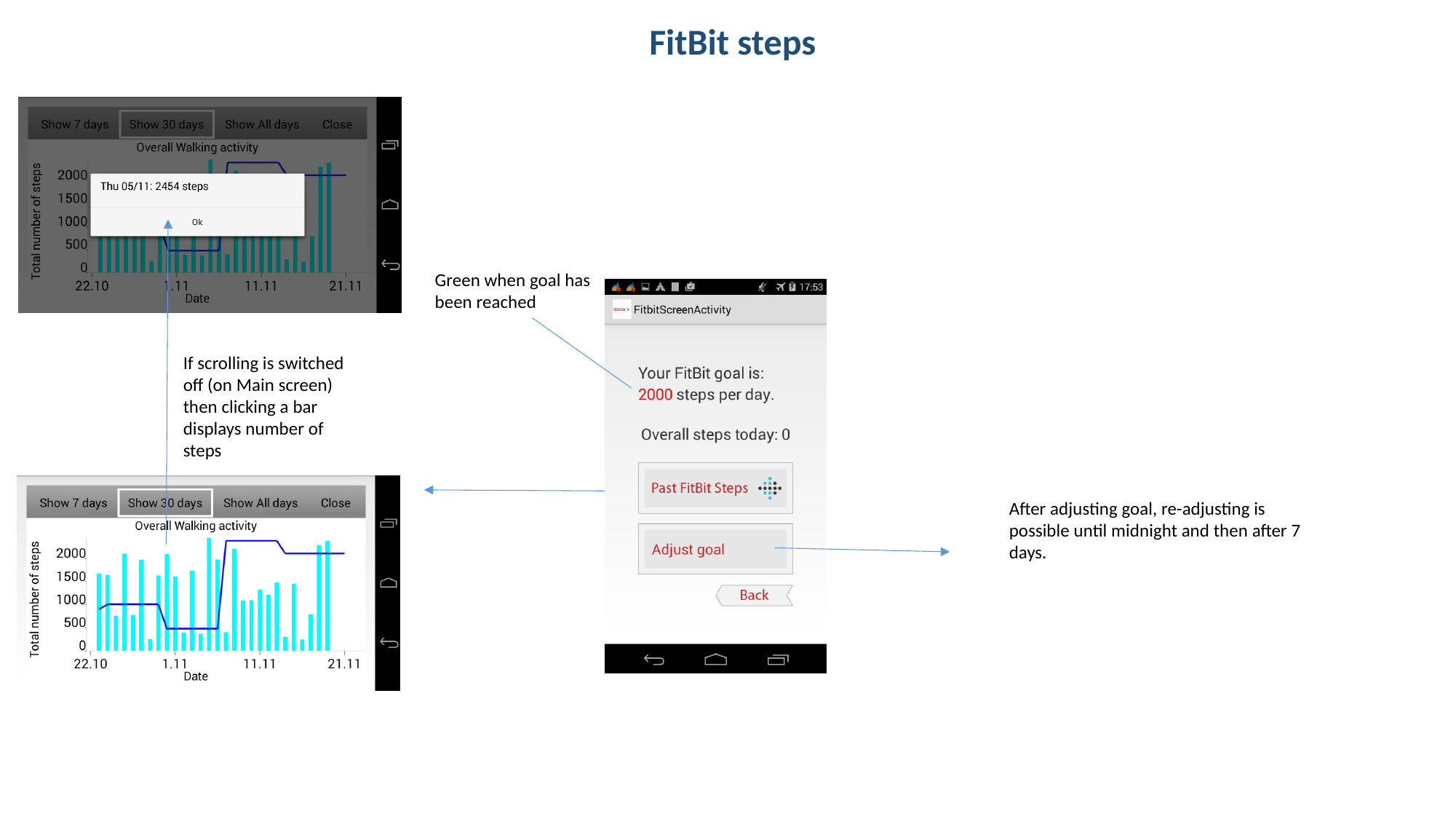

FitBit steps
Green when goal has been reached
If scrolling is switched off (on Main screen) then clicking a bar displays number of steps
After adjusting goal, re-adjusting is possible until midnight and then after 7 days.

## Slide 3
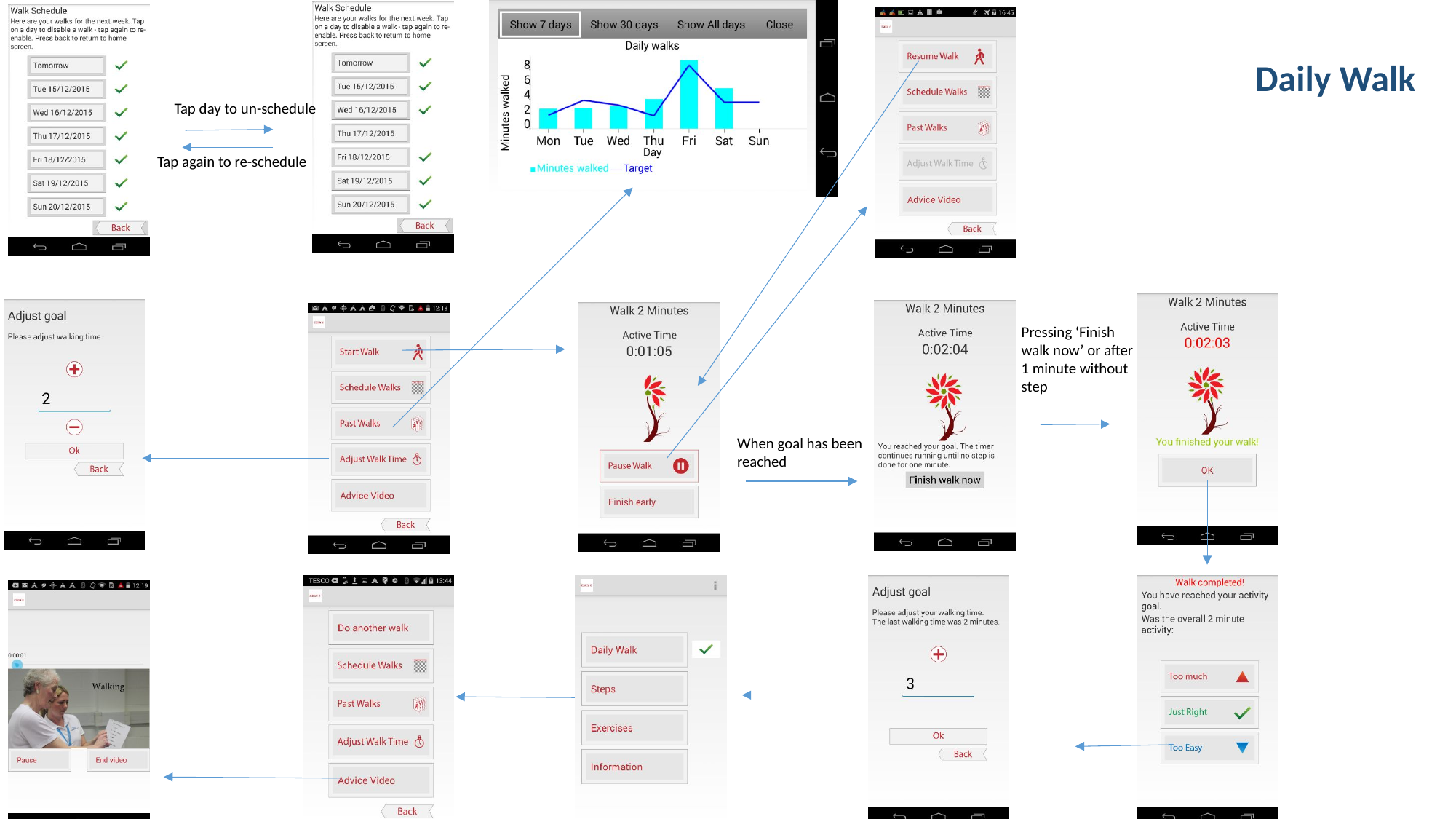

Daily Walk
Tap day to un-schedule
Tap again to re-schedule
Pressing ‘Finish walk now’ or after 1 minute without step
When goal has been reached

## Slide 4
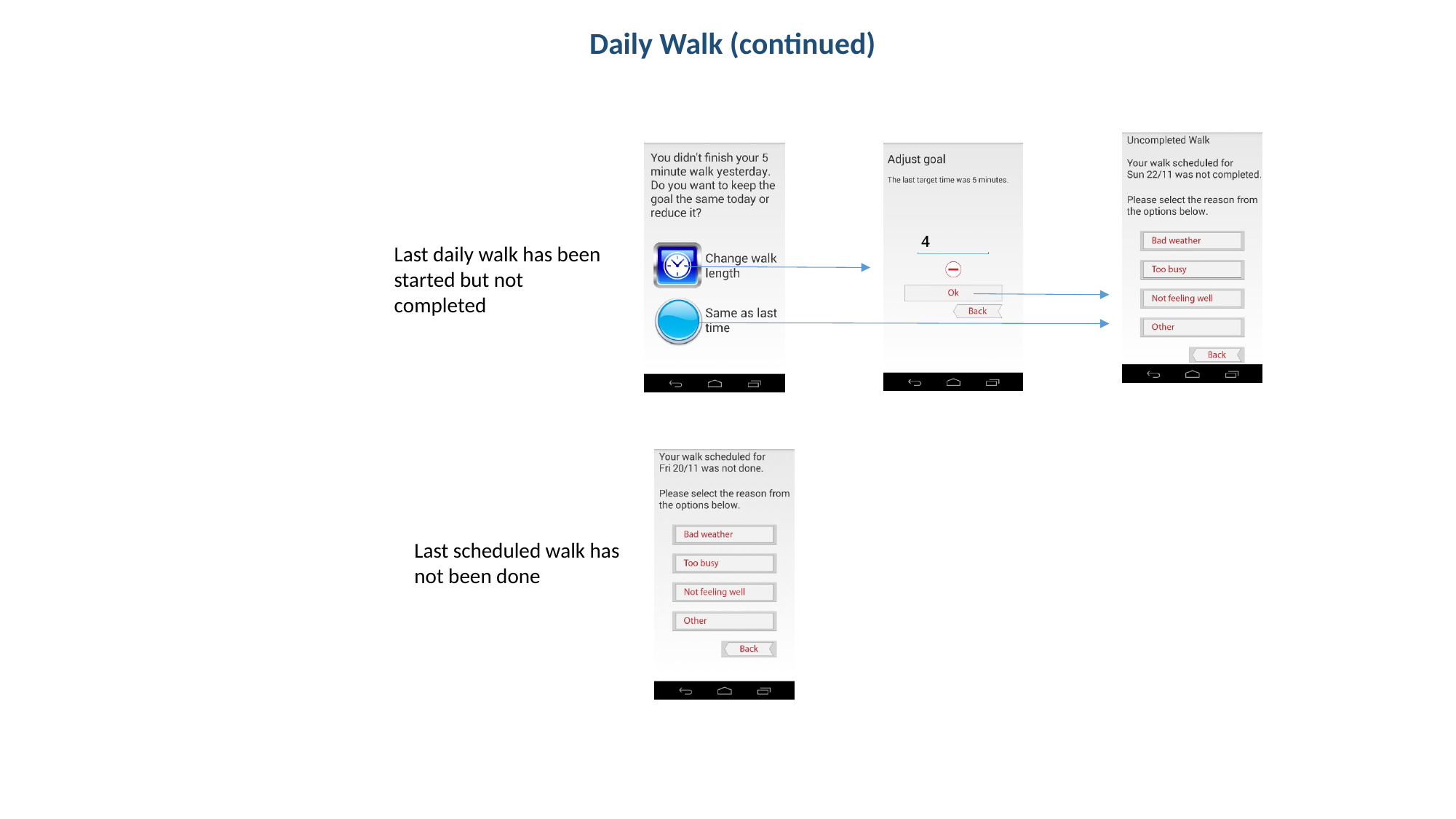

Daily Walk (continued)
Last daily walk has been started but not completed
Last scheduled walk has not been done

## Slide 5
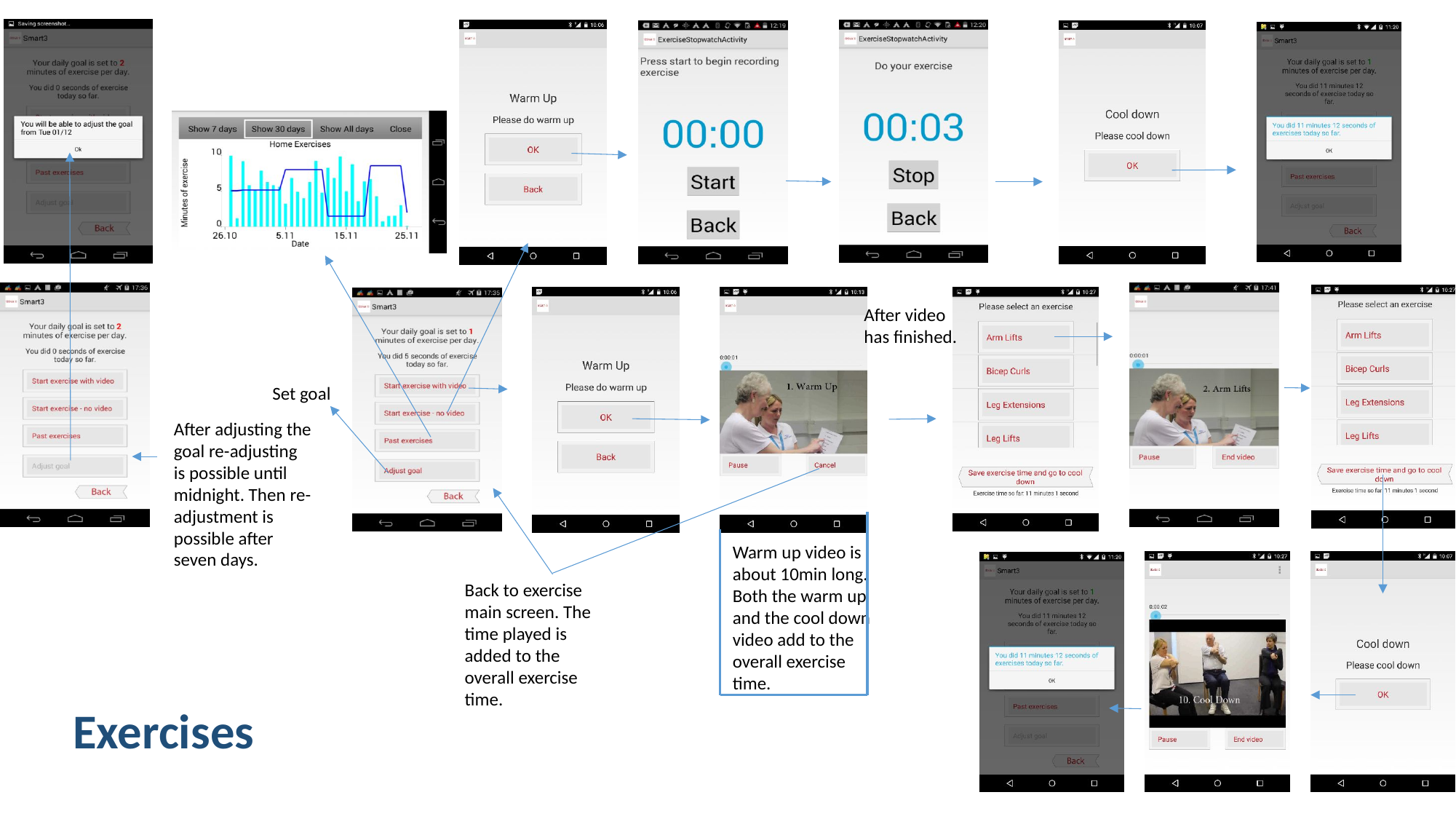

After video has finished.
Set goal
After adjusting the goal re-adjusting is possible until midnight. Then re-adjustment is possible after seven days.
Warm up video is about 10min long. Both the warm up and the cool down video add to the overall exercise time.
Back to exercise main screen. The time played is added to the overall exercise time.
Exercises
